# What Makes a Liveable Neighborhood? Role of Socio-Demographic, Dwelling, and Environmental Factors and Participation in Finnish Urban and Suburban Areas

**DOI:** 10.1007/s11524-024-00927-y

**Published:** 2024-10-24

**Authors:** Tytti P. Pasanen, Timo Lanki, Taina Siponen, Anu W. Turunen, Pekka Tiittanen, Vuokko Heikinheimo, Maija Tiitu, Arto Viinikka, Jaana I. Halonen

**Affiliations:** 1 Department of Public Health, Finnish Institute for Health and Welfare, Tampere, Finland; 2 Department of Public Health, Finnish Institute for Health and Welfare, Oulu, Finland; 3 Department of Public Health, Finnish Institute for Health and Welfare, Kuopio, Finland; 4 Department of Public Health, Finnish Institute for Health and Welfare, Helsinki, Finland; 5 Department of Environmental and Biological Sciences, University of Eastern Finland, Kuopio, Finland; 6 School of Medicine, University of Eastern Finland, Kuopio, Finland; 7 Finnish Environment Institute SYKE, Helsinki, Finland; 8 Department of Architecture, Aalto University, Espoo, Finland

**Keywords:** Urban planning, Neighbourhood satisfaction, Suburbs, Green space, Citizen participation, Socioeconomic deprivation

## Abstract

Quality of residential area, or neighborhood, is one determinant of overall quality of life and associated with health and health behavior. Ensuring that urban and suburban neighborhoods remain liveable and comfortable to reside in is a challenge amidst growing urbanization. Neighborhood quality has been associated with socio-demographic, dwelling-related, and subjective and objective neighborhood factors. However, only a few studies have additionally included participation in neighborhood decision-making and examined whether these associations are stronger among residents who are more settled in the neighborhood. We examined whether subjectively and objectively measured environmental factors and participation are associated with perception of neighborhood liveability and possible effect moderators. We analyzed survey data collected in 2021 among adults (aged 18–97) living in suburbs and urban centers in five Finnish cities (*n* = 2057; response rate 34%) with logistic regression models. Almost 80% of the respondents perceived their neighborhood as liveable or very liveable. The following factors were most strongly associated with perceiving the neighborhood as liveable: low area-level socioeconomic deprivation; central urban zone; green view from home; satisfaction with neighborhood safety, green areas, blue areas, and maintenance of traffic routes; and good or very good possibilities to influence decisions regarding the neighborhood. Only a few interactions between indicators of being more settled in the neighborhood (e.g., age group and dwelling ownership) modified these associations. In all, our study suggests that to maintain liveability, urban or community planning should consider local residents’ favorable perceptions of safety, recreational areas, and engagement in decision-making.

**Supplementary Information:**

The online version contains supplementary material available at 10.1007/s11524-024-00927-y.

## Introduction

Quality of living environment is one of the key aspects of perceived quality of life [1] and it has been found to predict mental health [2]. Half of the world’s population already live in urban areas, and this share is projected to reach 66% by 2050 [3]. The growing rate of urbanization makes cities subject to major changes in terms of increased number of residents, traffic flows, and other potential environmental stressors associated with urban living [4]. These issues pose a challenge for urban planning that should ensure that urban and suburban living environments remain liveable and support the well-being of their residents [5]. Urban planning solutions such as the presence of parks and commercial facilities can affect one’s emotional state, level of comfort, and physical activity patterns [6, 7]. Therefore, it is crucial to understand how different environmental attributes contribute to urban residents’ perceptions of their neighborhoods.

Finnish urban development has been characterized by rapid urbanization during the 1960s and 1970s [8]. Suburbs built at the time have had a negative reputation of having high level of social disorder and being unsafe [9]. Nevertheless, residents in these suburbs do not share these views, and in an international comparison, Finnish cities are safe [9]. On-going urbanization wave has pressured Finnish cities to densify suburbs, which can compromise their liveability [3, 10]. Hence, it is timely to assess what qualities in Finnish urban areas are being valued to ensure that urban and suburban living environments remain liveable from residents’ perspective.

Subjective evaluations of residential areas, or neighborhoods (used interchangeably here), have usually been assessed using the concept of neighborhood satisfaction [11] or liveability [12]. Liveability is a holistic concept, focussed on how the cultural and physical living environment support individual and community health and well-being [12, 13]. It is, thus, a similar but broader concept than environmental satisfaction, which generally refers to an individual’s attitude towards a specific place or its features [11]. In this study, accordingly, liveability was chosen as the global outcome reflecting subjective evaluation of the whole neighborhood, while satisfaction was used for assessing evaluations of specific environmental qualities within neighborhoods.

A recent meta-analysis found that 16% of the variation in overall neighborhood satisfaction and 7% of satisfaction with their specific components (such as relationships with neighbors or social disorder) can be explained by neighborhood-level differences [11]. This suggests that residents within the same neighborhood perceive their living environment and its attributes very differently and other factors may play a stronger role in neighborhood evaluations [11]. Factors identified in prior research are related to individual, dwelling, and environmental qualities [14].

Individual or household-level factors that predict neighborhood satisfaction include higher socioeconomic status indicating one has financial resources to choose one’s living environment [11]. Some demographic groups such as females and older people are on average more satisfied with their life in general and, in line, also with their neighborhoods [15, 16]. Moreover, other demographic characteristics such as living with a partner and/or children can reflect the degree of integration to the neighborhood and, accordingly, willingness to assimilate there [16].

Dwelling-related factors have also consistently correlated with neighborhood satisfaction [15, 17]. Owning one’s dwelling can indicate having placed more effort in selecting a suitable dwelling and neighborhood than when living in a rental dwelling, and it has been positively associated with greater neighborhood satisfaction [15, 18]. At the same time, being often more settled in the neighborhood, homeowners tend to be more sensitive to the neighborhood characteristics [18]. Other dwelling characteristics that have been found to play a role in neighborhood satisfaction include housing type [17] and natural views from home [19].

One of the key environmental predictors of neighborhood satisfaction has been safety, whether it be related to traffic or crime [15, 16, 20]. Safety is among the basic human needs and a prerequisite for actualizing other needs, related to social relationships and oneself, which may be supported by other environmental attributes. For example, good access to and size of green (e.g., urban parks) and blue spaces (e.g., coastal areas) and other recreational areas [14, 15, 19, 20], low levels of noise [14, 17], cleanliness, and overall pleasant appearance [14] have all been positively associated with neighborhood satisfaction.

In addition to these established correlates of neighborhood satisfaction, the role of community participation in decision-making has been highlighted as a crucial part of urban development to ensure cities are planned in the local context [12]. Participation in community decision-making has rarely been included in studies on neighborhood perceptions, although community participation is an increasingly recognized aspect of well-being and mental health, and it is one of the key aspects of European WHO Healthy Cities initiative [21]. Although tentative empirical evidence supports the view that participation in decision-making is associated with enhanced neighborhood satisfaction [22], a recent critical review concluded that liveability studies need to address community participation more broadly in different global contexts [12].

The present study assessed predictors of neighborhood liveability in urban and suburban areas of Finland, with two main research objectives. First, we were interested in the role of objective and subjective environmental factors as well as participation in neighborhood decision-making in neighborhood liveability evaluations. Second, we wanted to assess whether factors that might reflect being settled in the neighborhood moderate the association between the neighborhood environmental attributes and liveability. To achieve these, we controlled all analyses for established socio-demographic and dwelling-related confounders.

## Materials and Methods

### Study Areas and Sample

**Fig. 1:**
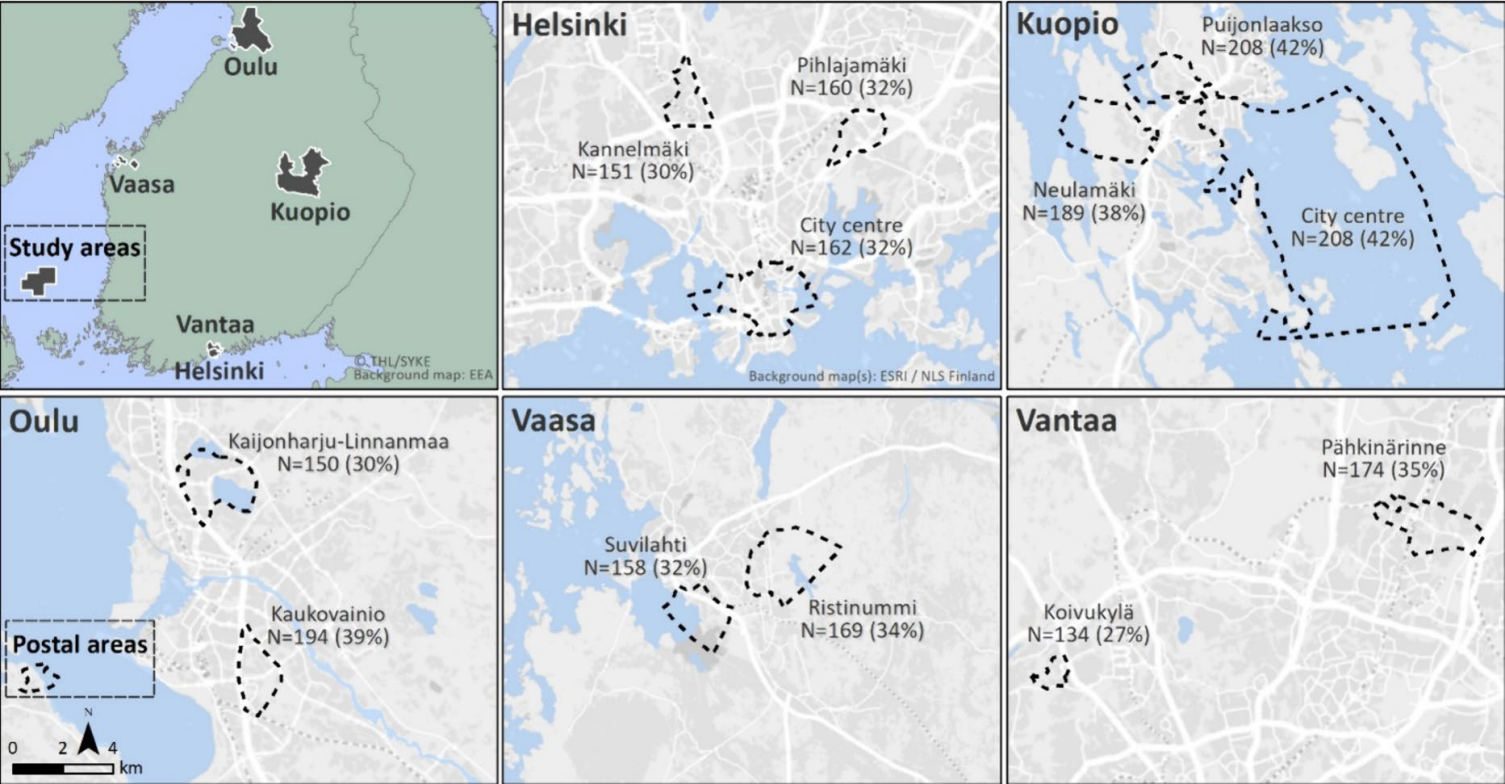
The locations of the postal areas included in the study and their response rates

An invitation to respond to the survey was sent by mail (including the paper-based questionnaire and a link to online questionnaire) to 6000 residents aged ≥ 18 years of two suburban postal areas in each of five Finnish cities (Helsinki, Vantaa, Kuopio, Vaasa, and Oulu), two city center postal areas in Kuopio, and six city center postal areas in Helsinki (Fig. 1). Residents were selected by simple random sampling by the Finnish Digital and Population Data Services Agency (official Finnish population registry). Each suburban postal area had a quota of 500 residents (max. one/household), and both city centers had a quota of 500 residents. The included cities are among the fifteen most populated cities in Finland, and their population in 2021 ranged from 68,000 (Vaasa) to 658,000 (Helsinki). All the suburban areas were dominated by apartment buildings and located a few kilometers from the city center. The first invitation was sent in October 2021, and after one reminder, 2072 questionnaires were returned by mid-December 2021. We excluded 15 questionnaires due to (1) duplicate responses (*n* = 6); (2) respondent having recently moved or living in other than the registered address (*n* = 6); and (3) the recipient not filling out the questionnaire themselves (*n* = 3). The final sample size was thus 2057 (response rate 34%).

### Factors Predicting Liveability

*Socio-demographic factors* included self-reported gender (male or female; “other” excluded due to < 30 responses, causing issues with convergence), age (in years), relationship status, having children in household, education, employment status, and annual household income. Categories for all predictors are shown in Table 1. *Dwelling factors* were also self-reported and included satisfaction with dwelling (on a scale 1 “very satisfied” to 5 “very dissatisfied”), type of building, ownership, living floor, and having a green view and the frequency of looking at it.

**Table 1:** Sample characteristics in the whole dataset and according to perceived neighborhood liveability

Variable	Category	Neighborhood evaluation					
		Total sample		Liveable or very liveable		Unliveable, very unliveable or neither	
		*n*	% or mean [SD]^a^	*n*	% or mean [SD]^a^	*n*	% or mean [SD]^a^
*Socio-demographic factors*							
Gender							
Female		1059	52.3	830	78.5	227	21.5
Male		966	47.7	731	76.0	231	24.0
Age in years		2025	46.5 [19.5]	1560	47.5 [19.5]	458	42.9 [19.0]
Relationship status							
Cohabiting with a partner		1058	52.6	866	81.9	191	18.1
In a relationship, not living together		233	11.6	165	71.1	67	28.9
Not in a relationship		721	35.8	520	72.5	197	27.5
Minors in the household							
No		1645	81.4	1248	76.2	390	23.8
Yes		377	18.6	309	82.0	68	18.0
Highest level of education							
Primary or lower secondary school		214	10.6	164	77.0	49	23.0
Upper secondary—vocational school		430	21.3	312	72.9	116	27.1
Upper secondary—general school		508	25.1	403	79.8	102	20.2
University or applied university		868	43.0	677	78.1	190	21.9
Employment status							
Employed		1000	49.4	785	78.5	215	21.5
Retired		518	25.6	415	80.7	99	19.3
Unemployed		135	6.6	94	70.7	39	29.3
Other (e.g., student, on parental leaves)		372	18.4	266	71.9	104	28.1
Annual household income							
< 15,000		415	21.2	283	68.5	130	31.5
15,000–30,000		469	24.0	365	78.2	102	21.8
30,001–50,000		456	23.4	363	79.8	92	20.2
> 50,000		612	31.4	493	80.6	119	19.4
*Dwelling-related factors*							
Dwelling ownership							
Owner-occupied		1010	50.1	833	82.7	174	17.3
Rental or others		1007	49.9	721	71.7	284	28.3
Type of dwelling							
Apartment		1540	76.4	1153	75.0	384	25.0
Row/terraced house		317	15.7	257	81.8	57	18.2
Detached house		160	7.9	143	89.4	17	10.6
Floor							
Ground floor (1st)		444	22.4	363	81.9	80	18.1
2nd		661	33.3	499	75.7	160	24.3
3rd or higher		880	44.3	662	75.5	215	24.5
Green view from home							
Yes, looking at it frequently		913	45.2	775	85.3	134	14.7
Yes, looking at it occasionally		608	30.1	467	76.9	140	23.1
No/yes but rarely looking at it		497	24.6	312	63.0	183	37.0
*Objective environmental factors*							
Urban zone							
Central pedestrian		319	15.7	270	85.2	47	14.8
Fringe of pedestrian		93	4.6	71	77.2	21	22.8
Intensive public transport		1076	53.2	799	74.4	275	25.6
Public transport		315	15.6	246	78.1	69	21.9
Car		221	10.9	174	79.1	46	20.9
Neighborhood socioeconomic deprivation index^b^		2025	0.5 [0.7]	1560	0.4 [0.7]	458	0.8 [0.7]
Green space coverage (%) within 1 km		2025	34.0 [16.1]	1560	34.1 [16.6]	458	33.8 [14.1]
Blue space coverage within 1-km							
< 0.1%		131	6.5	90	68.7	41	31.3
0.1–10%		1250	61.7	955	76.7	290	23.3
> 10%		644	31.8	515	80.2	127	19.8
*Subjective dwelling and environmental factors*							
Satisfied with dwelling							
No		325	16.1	149	45.8	176	54.2
Yes		1689	83.9	1403	83.4	279	16.6
Possibilities to influence neighborhood decisions							
Poor/very poor		621	31.2	406	65.5	214	34.5
Neither		842	42.3	696	82.8	145	17.2
Good/very good		254	12.8	228	90.1	25	9.9
Not interested		273	13.7	203	74.9	68	25.1
Satisfied with: road safety							
No		246	12.5	132	53.7	114	46.3
Yes		1727	87.5	1387	80.6	334	19.4
Other safety							
No		492	25.0	247	50.3	244	49.7
Yes		1479	75.0	1268	86.0	206	14.0
Noise level							
No		598	30.4	370	62.1	226	37.9
Yes		1370	69.6	1148	84.2	216	15.8
Public transport							
No		334	17.0	223	67.0	110	33.0
Yes		1638	83.0	1295	79.4	337	20.6
Street lighting							
No		312	15.8	198	63.7	113	36.3
Yes		1666	84.2	1323	79.7	337	20.3
Accessibility of traffic routes							
No		455	23.1	315	69.2	140	30.8
Yes		1512	76.9	1200	79.7	305	20.3
Maintenance of traffic routes							
No		550	28.0	344	62.7	205	37.3
Yes		1416	72.0	1170	83.0	240	17.0
Green spaces							
No		347	17.6	181	52.5	164	47.5
Yes		1627	82.4	1340	82.6	283	17.4
Blue spaces							
No		677	34.4	433	64.4	239	35.6
Yes		1293	65.6	1085	84.0	206	16.0
Community gardens etc							
No		1415	72.3	1054	74.8	356	25.2
Yes		541	27.7	453	83.7	88	16.3
Resting places outdoors							
No		1343	68.8	622	69.1	278	30.9
Yes		610	31.2	898	84.2	168	15.8
Cleanliness							
No		904	45.8	475	63.5	273	36.5
Yes		1068	54.2	1048	85.6	177	14.4
Outdoor meeting places							
No		751	37.9	704	69.6	308	30.4
Yes		1228	62.1	816	85.7	136	14.3
Outdoor art							
No		1016	51.6	1030	74.2	359	25.8
Yes		953	48.4	482	84.6	88	15.4

^a^% for categorical and mean [SD] for continuous variables

^b^An indicator based on area-level unemployment rate, educational attainment, and average income, ranging from − 1.43 to 2.69

*Objective environmental* factors included travel-related urban zones, reflecting both population density and transportation facilities [23] (Table 1). To define area-level socioeconomic deprivation, we used a summary score based on unemployment rate, educational attainment (proportion of those aged > 18 with primary or lower secondary education at highest), and household income (mean income proportional to the number of household, treated as additive inverse), calculated in 250 m × 250 m grids [24]. For each indicator, we derived a standardized *z*-score and used the mean value across all *z*-scores [25]. Higher values indicate higher socioeconomic deprivation.

Green spaces were based on CORINE land cover 2018 data from which we identified areas with a minimum size of 1.5 ha that are suitable for recreation [26]. Blue spaces comprised of lakes [27], rivers, and seas [28]. We used a 1-km buffer zone around each respondent’s home location which reflects recreational possibilities at the neighborhood-level within walking distance [29]. The share of blue spaces was highly skewed and, thus, collapsed into categories of none (< 0.1%), some (0.1‒10%), and more than 10%.

To assess *possibilities to influence neighborhood decisions*, the respondents were asked to rate their possibilities to influence the decisions regarding their residential area, on a scale 1 “very good,” 2 “good,” 3 “neither good nor poor,” 4 “poor,” 5 “very poor,” and 6 “not interested.” For the analyses, we combined options 1 and 2 (“good” or “very good”), 4 and 5 (“poor” or “very poor”), while “neither good nor poor” and “not interested” remained as separate categories. The question was designed by the project researchers.

*Subjective environmental factors* were asked with a question “How satisfied are you with the following aspects of your residential area?,” followed by a list of fourteen attributes (Table 1). The rating options were 1 “very satisfied,” 2 “satisfied,” 3 “neither satisfied nor dissatisfied,” 4 “dissatisfied,” and 5 “very dissatisfied.” Because responses 4 and 5 were rare, and to ease interpretation and model complexity, these were all recoded into binary variables reflecting whether the respondent was satisfied with the attribute (options 1 and 2) or not (options 3–5).

### Outcome

*Neighborhood liveability* was enquired by asking “In your opinion, how comfortable is your residential area?,” with the options 1 “Very comfortable,” 2 “Comfortable,” 3 “Neither comfortable nor uncomfortable,” 4 “Uncomfortable,” and 5 “Very uncomfortable.” Comfort and liveability have partially the same translation in Finnish (*viihtyisyys*). Environmental comfort refers to a holistic cognitive evaluation of the effect of surrounding environmental conditions on oneself [30]. Thus, it conceptually largely overlaps with liveability. Liveability has been more typically used in urban planning literature regarding neighborhoods (vs comfort in indoor conditions), and we therefore use the term “liveability” throughout this paper [13]. For the analyses, we categorized the outcome into a binary measure indicating whether the respondent found their residential areas as liveable (options 1 and 2) or not (options 3–5).

### Analytical Strategy

We first assessed descriptively the explanatory factors and neighborhood liveability. For the multivariate models, explanatory variables were added in the following steps, reflecting the literature on factors associated with neighborhood evaluations: (1) socio-demographic, (2) dwelling-related, (3) objective environmental factors, and (4) subjective dwelling and environmental factors, including participation in the decision-making process of the neighborhood. The model created in the last step (4) was considered our main model. Prior to the analyses, we screened the explanatory variables for multicollinearity using the scaled generalized variance inflated factor, suitable for both continuous and categorical variables, to ensure all values were below 2.5. We based the inference on effect sizes using odds ratios (OR) and their 95% confidence intervals (CI) and *p*-values (0.05 as the approximate threshold for “statistical significance”).

The multivariate models were specified with R software [31], using both a fixed effects and mixed models with a random intercept for every postal area. In the last analysis step, the model without random intercept showed a slightly better fit with the data based on Akaike’s and Bayesian information criteria, and hence, this approach was selected for the main models. Moreover, the outcome was initially specified as “ordinal,” but due non-convergence with including age and household income in the models, we specified the outcome as binary and checked that the results were consistent with the ordinal models. In all analyses, the sample was weighed to match the age and gender distributions in the target postal areas.

After building the main model, we assessed moderation by factors potentially reflecting more settled status in the neighborhood by adding one interaction term at a time for the following: having children, participation in decision-making, living with a partner, age (categorized into 18–34, 35–64, and 65 + years), dwelling ownership, and employment status (with categories with few cases merged), and those subjective and objective environmental factors that had the strongest association with neighborhood liveability (OR close to 2.00 or greater/0.50 or smaller).

### Sensitivity Analyses

We checked the robustness of our main results with several alternative model specifications. First, the main model was re-estimated in the following ways: without sample weights, adding a random intercept for the postal areas, specifying the outcome as ordered categorical (with probit link), and changing the threshold in the outcome to the highest category. Second, we re-ran the analyses using only the suburban sample (*n* = 1392) to see how much of the relationships was affected by urbanicity (the central urban sample was too small to run the final model on its own). Third, we used a 300-m buffer to assess green and blue space, reflecting recreational opportunities at almost immediate vicinity from home location. Fourth, we added only one subjective environmental factor at a time to the main model to get an idea how much their potential overlap might have affected their effects in the main model. Finally, we specified a model with all the perceived environmental factors without the objective environmental factors to investigate whether and how much the associations were affected by them.

## Results

### Descriptive

Most commonly the participants considered their neighborhoods as liveable (63.7%), whereas 13.7% considered them very liveable and 16.6% as neither. Only 5.3% rated their neighborhood as unliveable and 0.7% as very unliveable.

Regarding the environmental factors that can be influenced by urban planning, area-level socioeconomic deprivation was, on average, lower in neighborhoods rated as liveable and greater in unliveable neighborhoods (Table 1). The proportion of green space within a 1-km buffer from home, however, was very similar, approximately 34%, in both groups. The subjective environmental factors that differed the most between liveable and non-liveable neighborhoods were satisfaction with safety (both traffic and other) and satisfaction with green spaces (Table 1).

### Multivariate Models

In the main model (Table 2), the objective environmental factors that were associated with lower likelihood of perceiving the neighborhood as liveable vs not included living in the public transport zone (OR 0.48, *p* = 0.03) and socioeconomic deprivation (OR 0.51, *p* < 0.001). The percentage of green space within 1 km from home indicated higher likelihood prior to adding the subjective environmental factors (in model 3).

**Table 2:** Odds ratios (OR) and their 95% confidence intervals (CIs) in models 1–4. In bold: 95% CI does not overlap with 1

Variable	(Category)	Model 1 Socio-demographic factors (household/individual)		Model 2 Dwelling factors		Model 3 Objective environmental factors		Model 4 Subjective dwelling and environmental factors	
		OR	95% CI	OR	95% CI	OR	95% CI	OR	95% CI
Age in years (continuous)		**1.02**	[1.01; 1.03]	1.01	[1.00; 1.02]	1.01	[1.00; 1.02]	**1.02**	[1.01; 1.04]
Male gender (vs female)		0.87	[0.70; 1.08]	0.91	[0.73; 1.15]	1.10	[0.85; 1.43]	1.05	[0.79; 1.41]
Relationship status (ref. cohabiting with a partner)									
In a relationship, not living together		**0.68**	[0.48; 0.98]	0.71	[0.49; 1.04]	0.82	[0.54; 1.25]	0.76	[0.47; 1.23]
Not in a relationship		**0.64**	[0.49; 0.84]	**0.72**	[0.55; 0.95]	**0.69**	[0.51; 0.94]	**0.61**	[0.42; 0.86]
Minors in the household (yes/no)		1.23	[0.90; 1.70]	1.11	[0.80; 1.54]	1.42	[0.98; 2.07]	1.41	[0.94; 2.13]
Highest education (ref. primary or lower secondary school)									
Upper secondary—vocational		0.79	[0.51; 1.21]	0.78	[0.50; 1.22]	0.77	[0.46; 1.27]	0.85	[0.46; 1.54]
Upper secondary—general		1.38	[0.88; 2.15]	1.22	[0.77; 1.92]	1.11	[0.66; 1.83]	1.01	[0.56; 1.83]
University or applied university		1.09	[0.70; 1.68]	1.00	[0.64; 1.56]	0.88	[0.53; 1.44]	0.85	[0.47; 1.52]
Employment status (ref. employed)									
Retired		0.72	[0.45; 1.13]	0.71	[0.44; 1.14]	0.62	[0.36; 1.05]	0.58	[0.31; 1.09]
Unemployed		0.87	[0.56; 1.39]	0.88	[0.55; 1.42]	1.04	[0.61; 1.79]	1.31	[0.71; 2.48]
Other (e.g., student, on parental leaves)		1.03	[0.72; 1.48]	1.04	[0.73; 1.51]	0.91	[0.6; 1.37]	0.95	[0.60; 1.52]
Annual household income € (ref. < 15,000)									
15,000–30,000		1.36	[0.97; 1.92]	1.33	[0.94; 1.89]	**1.56**	[1.04; 2.35]	**2.00**	[1.26; 3.20]
30,001–50,000		1.30	[0.89; 1.89]	1.19	[0.80; 1.77]	1.20	[0.77; 1.87]	1.48	[0.89; 2.48]
> 50,000		1.19	[0.79; 1.77]	1.05	[0.68; 1.62]	0.89	[0.54; 1.45]	1.12	[0.64; 1.98]
Rental/other (vs owner-occupied) dwelling				0.83	[0.62; 1.11]	1.16	[0.84; 1.61]	1.19	[0.82; 1.74]
Type of dwelling (ref. apartment)									
Row/terraced house				1.32	[0.92; 1.91]	1.13	[0.75; 1.72]	1.27	[0.79; 2.05]
Detached house				1.55	[0.87; 2.90]	1.03	[0.54; 2.05]	0.97	[0.45; 2.15]
Living floor (ref. ground/first)									
2nd				0.86	[0.62; 1.20]	0.96	[0.66; 1.40]	0.94	[0.60; 1.45]
3rd or higher				0.93	[0.66; 1.3]	0.87	[0.59; 1.27]	0.87	[0.55; 1.36]
Green view from home (ref. no/yes but rarely looking at it)									
Yes, looking at it frequently				**2.85**	[2.16; 3.79]	**2.64**	[1.91; 3.65]	**2.14**	[1.48; 3.10]
Yes, looking at it occasionally				**1.80**	[1.36; 2.37]	**1.85**	[1.35; 2.53]	**1.47**	[1.03; 2.12]
Urban zone (ref. central pedestrian)									
Fringe of pedestrian						0.64	[0.32; 1.30]	0.53	[0.24; 1.20]
Intensive public transport						0.60	[0.36; 1.00]	0.71	[0.38; 1.30]
Public transport						**0.48**	[0.28; 0.83]	**0.48**	[0.25; 0.91]
Car						**0.48**	[0.26; 0.90]	0.53	[0.25; 1.10]
Neighborhood socioeconomic deprivation						**0.45**	[0.35; 0.57]	**0.51**	[0.38; 0.67]
Blue space within a 1-km buffer (ref. < 0.1%)									
0.1–10%						0.99	[0.60; 1.61]	0.80	[0.45; 1.38]
> 10%						1.05	[0.61; 1.79]	0.77	[0.41; 1.43]
Green space (%) within a 1-km buffer						**1.14**	[1.03; 1.27]	1.09	[0.97; 1.23]
Satisfied with dwelling (vs not)								**3.88**	[2.74; 5.51]
Possibilities to influence neighborhood decisions (ref. poor/very poor)									
Good/very good								**3.20**	[1.75; 6.15]
Neither								**1.58**	[1.13; 2.19]
Not interested								1.35	[0.87; 2.11]
Satisfied with factors in the residential area (vs not)									
Road safety								1.49	[0.98; 2.26]
Other safety								**2.95**	[2.11; 4.14]
Noise level								1.16	[0.83; 1.61]
Public transport								0.99	[0.68; 1.44]
Street lighting								1.14	[0.76; 1.71]
Accessibility of traffic routes								**0.61**	[0.42; 0.88]
Maintenance of traffic routes								**2.09**	[1.49; 2.93]
Green spaces								**1.91**	[1.31; 2.79]
Blue spaces								**1.85**	[1.32; 2.60]
Community gardens etc								0.86	[0.59; 1.25]
Resting places outdoors								1.27	[0.93; 1.74]
Cleanliness								1.35	[0.99; 1.84]
Outdoor meeting places								1.22	[0.87; 1.72]
Outdoor art								1.37	[0.95; 1.98]
Model pseudo *r*^2^ (Nagelkerke)			0.13	0.20		0.39		0.57	

Regarding the subjective environmental and dwelling factors, being satisfied with dwelling (OR 3.88, *p* < 0.001), general safety (OR 2.95, *p* < 0.001), maintenance of traffic routes (OR 2.09, *p* < 0.001), green spaces (OR 1.91, *p* = 0.001), and blue spaces (OR 1.85, *p* < 0.001) were associated with greater odds of perceiving the neighborhood as liveable versus not. Satisfaction with accessibility of traffic routes was associated with lower odds of perceiving the neighborhood as liveable (OR 0.61, *p* = 0.001). Moreover, the odds of perceiving the neighborhood as liveable was greater among those who rated their possibilities to influence neighborhood decisions as good or very good (OR 3.20, *p* < 0.001) or neither good nor poor (OR 1.58, *p* = 0.007), compared with those with poor possibilities.

Of the socio-demographic factors, each 1-year increase in age and one of the higher income categories were associated with greater odds of perceiving the neighborhood as liveable (vs not). Compared with those living with a partner, those not in a relationship perceived their neighborhood as liveable less likely. In terms of the dwelling factors, having a green view and the frequency of looking at it was associated with greater probability of perceiving the neighborhood as liveable versus not.

### Effect Modification

The environmental factors for which the effect modification by being more settled in the neighborhood was tested (using interaction terms) were satisfaction with general safety, maintenance of traffic routes, green spaces, and blue spaces; area-level socioeconomic deprivation; and urban zone (re-categorized into three groups to obtain sufficient sample per group).

No interaction effects were present for these environmental factors and having children, dwelling ownership status, or possibilities to influence neighborhood decisions (with this, the interaction model between urban zone did not converge).

**Fig. 2:**
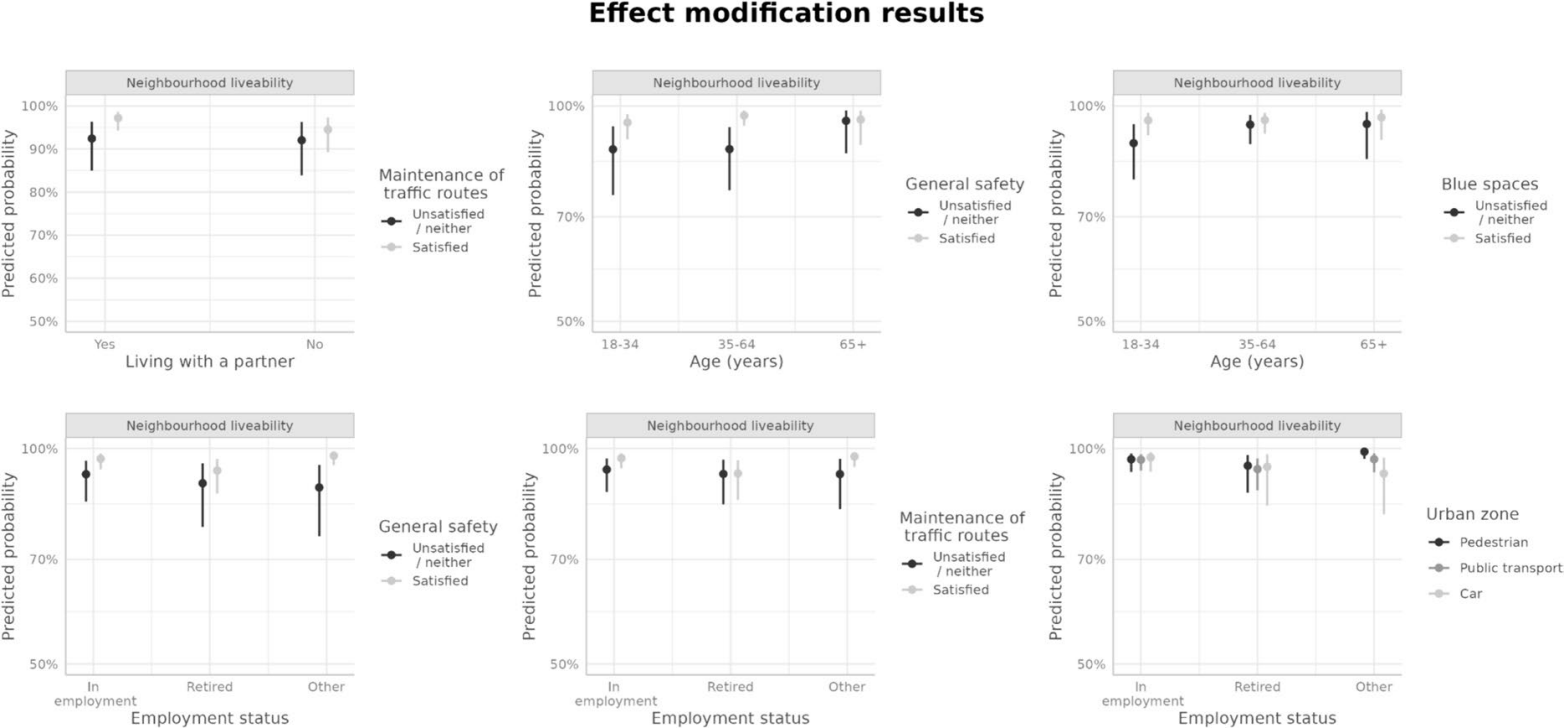
Predicted probabilities of neighborhood liveability in effect modification analyses (calculated at the median values for continuous and mode values for categorical predictors)

Other interactions were mostly observed for age groups and employment status, but they had wide confidence intervals (Fig. 2). For age, the interaction term for comparing those aged 65 or more to 18–34 years in terms of satisfaction with safety was 0.40 (*p* = 0.03). The interaction term comparing those aged 35–64 with those aged 18–34 in terms of satisfaction with blue space was 0.50 (*p* = 0.03). Compared with employed respondents, among the unemployed and other (parental leaves or students), satisfaction with safety was associated with higher odds of finding the neighborhood as liveable (OR_interaction_ 2.2, *p* = 0.03), whereas living in either public transportation (OR_interaction_ 0.30, *p* = 0.03) or car-intensive (OR_interaction_ 0.10, *p* = 0.001) urban zone was associated with lower odds. Among retired, compared with employed, the interaction term for the maintenance of traffic routes was lower, 0.46 (*p* = 0.04). Finally, compared with respondents who lived with a partner, among those who did not, the maintenance of traffic routes (OR_interaction_ 0.53, *p* = 0.03) was associated with lower odds of neighborhood liveability.

### Sensitivity Analyses

We observed no changes in the main results in the following sensitivity analyses: model including random intercept for postal area, unweighted sample, outcome specified as ordered categorical, outcome threshold changed into “very liveable” vs other (some differences in the socio-demographic measures’ estimates were observed though), and omitting the objective environmental factors.

Only a few results changed with different variable selection or specification. In the model restricted to the suburban respondents, satisfaction with outdoor art was also associated with increased odds of neighborhood liveability (OR 1.57, *p* = 0.04), but satisfaction with accessibility of traffic routes was not (OR 0.82, *p* = 0.37). In the model with 300-m buffer for green and blue space, having more than 10% of blue space resulted in a positive association with liveability (OR = 2.17, *p* = 0.03). When adding the subjective environmental factors one at the time, all showed a positive association (Supplementary Fig. A1). Moreover, in many of these models, green space showed a positive association with neighborhood liveability.

## Discussion

We examined a range of subjective and objective environmental factors in relation to neighborhood liveability in suburban and urban areas of Finland, controlling for socio-demographic and dwelling characteristics. Of perceived environmental factors, satisfaction with general safety was most strongly associated with perceiving one’s neighborhood as liveable, which supports many previous studies showing greater neighborhood satisfaction with better safety [15, 16, 20, 32]. Satisfaction with general safety was followed by satisfaction with maintenance of traffic routes, relevant especially in the Nordic countries where sanding and snow clearance are needed during winter months when our survey was partially collected. In addition, different indicators of nature near home—green views, satisfaction with green and blue space, and, to some extent, the coverage of green spaces around home—were among the strongest environmental predictors of liveability in this study. Having good possibilities to participate in neighborhood decision-making, a novel predictor in the neighborhood satisfaction literature, was also a strong predictor of neighborhood liveability. Finally, we assessed effect modification between the strongest environmental factors and potential indicators of a more settled status in the neighborhood but found few consistent patterns.

The results showing that different measures of natural areas, including green views from home and satisfaction with green and blue spaces, were strongly and consistently associated with neighborhood liveability are not surprising, given the accumulating evidence on their beneficial role for human health and well-being [33]. Consistent with previous studies on neighborhood satisfaction [20], residents’ perceived satisfaction with green and blue spaces in the neighborhood was more strongly associated with neighborhood liveability than the proportion of green and blue spaces around residence. Interestingly, different measures of green space—views from home, proportion of green spaces in the residential area, and satisfaction—did not show major overlap in their relationship with neighborhood liveability. It seems that various aspects of natural environments can independently contribute to the perception of neighborhood liveability.

Regarding the objective environmental qualities, lower socioeconomic deprivation of the neighborhood and central urban zone were consistently associated with greater odds of neighborhood liveability. Similar to our results, Baum et al. [34] found that regardless of household income, the share of low-income households in the neighborhood was associated with lower, and the share of high-income households with greater neighborhood satisfaction. In our study, the association for area-level deprivation was evident even when controlling for a range of other subjective and objective environmental factors. Neighborhood socioeconomic deprivation was the lowest in the more central urban zones, where also neighborhood liveability was more common than in the zones further from central locations.

Having good possibilities to participate in neighborhood decision-making was one of the strongest predictors of neighborhood liveability. Similar results, emphasizing different forms of community participation for neighborhood satisfaction, have been previously observed [22]. Engaging residents has been found to be particularly important for residents’ well-being in urban infill developments [10], which is relevant also in Finland with increasing rates of urbanization [3]. It is noteworthy that only 13% of all respondents rated their possibilities to influence neighborhood decisions as good or very good. A follow-up question is, then, how to improve participation opportunities. Participation in local decision-making can take many forms such as being involved in the city planning, local politics, neighborhood associations, or participatory planning. Cities can better engage residents in planning processes by collecting opinions digitally or in person using diverse methods including workshops, focus groups, social media, or polls [35]. In our study, we had no information on the different types of participation respondents had engaged in or how they would have wanted to participate. Nevertheless, given the strong association with neighborhood liveability, more detailed investigations on types of participation and lessons-learned in the Finnish context are needed in prospective studies.

Overall, we found limited evidence supporting the idea that neighborhood environmental factors would be stronger predictors of perceived neighborhood liveability among those who are more settled in the area [11]. None of the strongest environmental predictors for neighborhood liveability showed interactions with dwelling ownership, having children in the household, or participation in neighborhood decision-making. Satisfaction with maintenance of traffic routes was associated with greater odds of neighborhood liveability for employed versus retired and those living with a partner (versus not). The difference between employed and retired could be related to regular commuting that requires a good level of maintenance during the Nordic winters. Interestingly, satisfaction with safety seemed to be most strongly associated with neighborhood liveability among young adults (aged 18–34 versus 65 or more) and those who were either unemployed, students, or on parental leaves. These associations could be explained by different patterns of use of the neighborhood between different age groups. For example, younger people are more likely students than older age groups, and they may move within the neighborhood at later times of the day than older people, when safety is generally a greater concern.

To our knowledge, our study was the first to assess the importance of possibilities to participate in neighborhood decision-making for neighborhood evaluations in Finnish suburban context, while controlling for socio-demographic, dwelling, and environmental characteristics. Yet, it has some limitations. First, majority of the environmental factors were perceived measures, which limits their direct application to urban and community planning. Second, previous studies have shown that ethnic diversity might be a relevant factor for neighborhood satisfaction, but we had no measure on this on either individual or area level. Third, the question on participation in decision-making was not validated, and it is possible that respondents interpreted it in different ways. Finally, this study was cross-sectional, and we cannot infer whether the factors that showed the strongest relationship with neighborhood liveability are its causal predictors. To assess this question, we encourage longitudinal or quasi-experimental study designs.

## Conclusion

Taken together, our study suggests that in urban and apartment-dominated suburban areas of Finland, liveability is most strongly associated with satisfaction with safety, maintenance of traffic routes, and green and blue spaces in different forms, as well as with good opportunities to participate in decision-making in the neighborhood. These aspects are, therefore, important to consider in any urban or community development plans. Although the majority of the respondents perceived their neighborhoods as liveable and were satisfied with their specific attributes, special attention should be paid to socioeconomically deprived areas where liveability ratings were consistently lower.

## Supplementary Information

Below is the link to the electronic supplementary material.

Supplementary file1 (DOCX 125 KB)

## Data Availability

Due to restrictions stated in the privacy notice, the individual level data cannot published as open access nor be provided to researchers outside the research group. Those objective environmental factors that do not contain sensitive information (that is, all except for neighnourhood-level socio-economic deprivation) are available as open data. More information on specific layers via https://ckan.ymparisto.fi/en/.
