# Supplementary material for: What Makes a Liveable Neighborhood? Role of Socio-Demographic, Dwelling, and Environmental Factors and Participation in Finnish Urban and Suburban Areas
Source: J Urban Health. 2024 Oct 24;101(6):1207–20. doi: 10.1007/s11524-024-00927-y (PMC11652550; doi:10.1007/s11524-024-00927-y)
Supplement: Supplementary file 1 — Supplementary file1 (DOCX 125 KB) [file 11524_2024_927_MOESM1_ESM.docx]

**
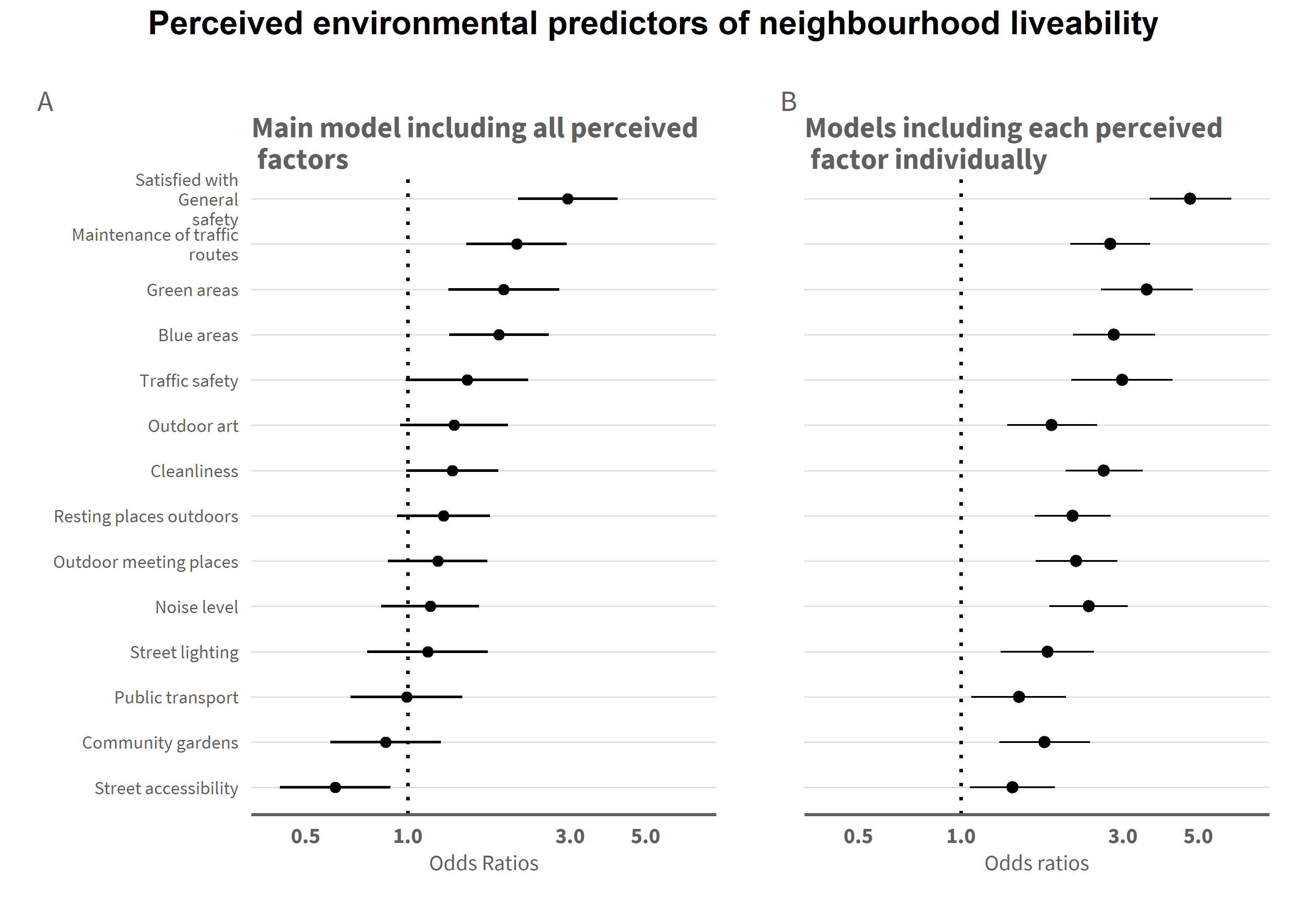
**

**Supplementary Fig. A1** Effect estimates for neighbourhood liveability in relation to perceived satisfaction with environmental factors in A) the main model and B) in sensitivity models where each subjective environmental factor was tested one at a time, controlling for all socio-demographic, dwelling, and objective environmental factors
